# Integrating music and nature: a scoping review of research on interventions involving both music- and nature-based strategies for mental health and wellbeing

**DOI:** 10.3389/fnhum.2025.1664304

**Published:** 2025-08-26

**Authors:** Michelle D. Hand, Emily S. Ihara, Morgan Moore, Madison Shaw

**Affiliations:** Department of Social Work, College of Public Health, George Mason University, Fairfax, VA, United States

**Keywords:** music, nature, scoping review, qualitative, thematic analysis

## Abstract

**Introduction:**

Both music-related and nature-based therapeutic activities can enhance wellbeing, physical, social, emotional, and mental health, and recovery from posttraumatic stress. While music- and nature-based therapeutic approaches have been studied individually, research is limited on the holistic combination of music- and nature-based therapeutic interventions. Thus, a scoping review was conducted to chart primary research on the combined use of music- and nature-based therapeutic strategies and their effects on overall wellbeing, and within this scope, potentially on mental and behavioral health outcomes.

**Methods:**

Included were peer-reviewed articles reporting primary research findings on how (if at all) the combined use of both music and nature-based interventions impacted wellbeing and thus, mental and behavioral health. All studies had to be reported in English. Excluded were studies that did not involve both nature-based and music-related therapeutic activities, those not involving primary research, and articles without a clear discussion on potential impacts on wellbeing, mental, or behavioral health. After applying this inclusion and exclusion criteria, 884 potentially relevant peer-reviewed articles were identified, 23 of which were preliminarily screened in upon abstract and title review. After full text reading, eight of these articles were deemed eligible for the review and were thematically analyzed.

**Results:**

Four themes were identified from the reviewed studies: (a) music- and nature-based activities yield benefits across various aspects of wellbeing, (b) multiple activities can be combined and adapted for diverse contexts and populations, (c) more research is needed on the combined therapeutic use of music and nature, and (d) choice and expression should be prioritized, which music and nature can facilitate. The primary focus of the reviewed studies was on how music-based interventions in outdoor or natural settings can impact multiple aspects of wellbeing, particularly emotional wellbeing, and with this, improved mood.

**Discussion:**

The results suggest that combining music and nature-based therapeutic approaches can improve mental and behavioral health by enhancing multiple aspects of wellbeing. For example, music-making in natural settings can foster deep connections with nature and spiritual wellness. Implications for future research are provided, as further research is needed on the combined use of music- and nature-based therapeutic activities.

## Introduction

The established need for holistic, accessible and effective interventions to support mental health and wellness has gained significant attention in recent years, especially with the global rise of depression and anxiety (World Health Organization [WHO], 2022). Traditional methods of addressing mental health including talk therapy and medication may be costly and unsuitable for everyone, as one size, or a single traditional intervention, does not fit all (van der Kolk, 2015). As a result, there is a growing demand for exploring alternative approaches that are more holistic, affordable, and accessible to the broader public (Seetharaman et al., 2021).

Both music and nature-based therapeutic activities have independently demonstrated positive effects on mental health and wellbeing (Jimenez et al., 2021; Kumpulainen et al., 2025). As Oliver Sacks (2019), a renowned neurologist famously reflected, “in 40 years of medical practice, I have found only two types of non-pharmaceutical “therapy” to be vitally important for patients with chronic neurological diseases: music and gardens,” (p. 243). Research has supported this observation; both music (Rebecchini, 2021) and time in or engaging with nature (Nejade et al., 2022) have been shown to impact emotions, increase relaxation, reduce stress, and enhance overall mood (While, 2020; Kumpulainen et al., 2025). As such, music and nature-based strategies have increasingly been studied as potentially promising therapeutic strategies to enhance physical, mental, and behavioral health, wellbeing, recovery from trauma, along with connections with others, with one’s environment, and with culture (Pfeifer et al., 2019; Sacks, 2019; Jimenez et al., 2021; Rebecchini, 2021; Nejade et al., 2022; Kumpulainen et al., 2025).

Music interventions engage multiple brain regions and simultaneously activate multisensory factors including auditory, visual, motor, memory, attention, and emotion information (Degé and Kerkovius, 2018; Sutcliffe et al., 2020). Such multisensory activation may create changes in the brain, by strengthening existing neural connections as well as creating new neural links (Chatterjee et al., 2021). Regular engagement with music has also been associated with improvements in executive functioning, memory, and attention, suggesting potential protective effects against cognitive decline (Sarkamo et al., 2012; Seinfeld et al., 2013; Chaddock-Heyman et al., 2021). At the behavioral level, music influences neurochemical activity—including dopamine, serotonin, and oxytocin—thereby supporting stress regulation, emotional balance, and social connectedness (Chanda and Levitin, 2013).

Given these overlapping benefits, leveraging music and nature together may offer a synergetic intervention with significant therapeutic potential. As such, music has been incorporated in existing therapeutic farm, or care farms (Huis In Het Veld et al., 2015). However, research on their synergistic application for improved mental health among participants is limited; research is still emerging on the combined use of music- and nature-based therapeutic interventions for mental health (Pfeifer et al., 2019; Kumpulainen et al., 2025).

This scoping review is underpinned by a contemporary trauma-informed care approach, which involves normalizing trauma and underscoring needs for accessible and culturally relevant interventions (Goodwin and Tiderington, 2022; Hand, 2024). As such, consideration was given to the embodied nature of trauma and the importance of developing, implementing, and evaluating interventions that include bilateral stimulation as well as opportunities for enhanced awareness and control of one’s body (Bensimon et al., 2008; Rhodes, 2015; van der Kolk, 2015; van de Kamp et al., 2019), which can improve wellbeing and facilitate recovery from residual embodied trauma (Clarkson and Cavicchia, 2014; Ruzany, 2022).

An example of bilateral stimulation through music is drumming, which can engage both sides of the brain by using both arms and at times, legs, to produce musical sounds (Amad et al., 2016). This can result in more control over one’s body (Bensimon et al., 2008). Such interventions, using bilateral stimulation, which can include horticulture-based activities, such as planting and engaging with nature, and can result in healing from traumatic events, which are generally logged in the non-speaking side of the brain (van der Kolk, 2015). The effectiveness of talk therapy is limited for resolving trauma, making a case for interventions that do not require talking yet encourage bilateral engagement (van der Kolk, 2015).

Both music making and nature-based activities often involve bilateral stimulation and can activate the autonomic nervous system by lowering stress levels and encouraging physiological states that are associated with relaxation and recovery (Corbijn van Willenswaard et al., 2017; Lorber and Savič, 2011; Sudimac et al., 2022). Music-based strategies can achieve this through rhythmic synchronization, deep breathing, and promoting social connection (Corbijn van Willenswaard et al., 2017), while nature-based interventions can lower heart rate and engage the autonomic nervous system by offering calming stimuli, which can result in deep respiration, mindful engagement, and connection to others as well as to one’s environment (Detweiler et al., 2015; Corbijn van Willenswaard et al., 2017; Sudimac et al., 2022). Thus, music- and nature-based activities can be used to help the body shift away from the stress-driven sympathetic state, promoting balance and wellbeing (Detweiler et al., 2015; Corbijn van Willenswaard et al., 2017; Sudimac et al., 2022). Engagement with sensory stimuli, such as auditory stimuli from music and multisensory experiences while in or interacting with nature has been shown to reduce stress and improve mood (Arbuthnott and Sutter, 2019). Still, further research is needed on the combined use of music and nature to enhance wellbeing (Adams and Beauchamp, 2019).

What follows is a scoping review that was completed to explore the impact of combining music and nature as an effective intervention to improve wellbeing, and with this, mental health and/or behavioral health outcomes. To achieve this objective, the specific aims were to identify and summarize existing peer-reviewed research published on this topic with a focus on studies that examine the effects of music and nature on wellbeing and within this scope, potential mental health and behavioral health outcomes. The second aim of this scoping review was to identify themes and potential gaps in extant peer-reviewed literature to offer meaningful implications for future research and for future therapeutic programming involving music- and nature-based activities.

## Materials and methods

As opposed to systematic reviews, which synthesize research that has been well-established, often through randomized control trials (e.g., on adverse childhood experiences), scoping reviews are conducted when research topics are still emerging and thus, have not been extensively examined or when research is complex in nature (Hand et al., 2022). Considering the limited extant research on the combined therapeutic use of music and nature, a scoping review was conducted. This article offers a summary of the results of a scoping review focused on the current state of peer-reviewed primary research on the combined therapeutic use of music and nature and how (if at all) this combined use of music and nature may impact wellbeing and with this, potential mental health and behavioral health outcomes.

The overarching research question in for this scoping was: “What primary scholarly research has been conducted to date on the combined therapeutic use of music and nature for improving wellbeing, and with this, potential mental and behavioral health outcomes?” Owing to the anticipated limited extant research on the combined therapeutic use of music and nature, no parameters were identified surrounding the population of interest; the combined use of music- and nature-based interventions were explored across populations, with no comparators, and a focus on potential effects on wellbeing, and thus, on mental and behavioral and health outcomes.

### Conceptual framing

This work was guided by an integrated theoretical framework that draws from the Biophilia Hypothesis (Wilson, 1993; Kellert, 1993), Polyvagal Theory (Porges, 2011), and Embodiment Theory (Csordas, 1994). Ecopsychology explores the emotional link and interdependence between human beings and nature as well as the natural environment in consideration of the Biophilia Hypothesis, which suggests that people are deeply drawn to nature not only for growing or finding food or medicinal herbs, but also for their overall wellbeing (Wilson, 1993, Kellert, 1993; Montgomery and Courtney, 2015). Thus, ecopsychology explores the human desire for nature for its aesthetics as well as for various aspects of wellness (e.g., emotional, cognitive, intellectual, and spiritual) with an understanding that people are biophilic or naturally drawn to plants and their natural environment (Wilson, 1993). As such, research has begun to explore the use of nature-based activities for mental health and wellbeing (Ulrich, 1993; Montgomery and Courtney, 2015) along with the integration of music in nature-based spaces (Adams and Beauchamp, 2019).

Poloyvagal Theory (Porges, 2011) and Embodiment Theory (Csordas, 1994) together highlight the importance of regulating the autonomic nervous system and acknowledging the role of the body in processing trauma. Polyvagal Theory emphasizes the role of rhythmic, relational, and sensory experiences, such as drumming or immersion in nature, in activating the parasympathetic nervous system and supporting neurophysiological states of safety and connection (Porges, 2011). Embodiment Theory further underscores the significance of engaging the body as a site of lived experience and healing, aligning with the somatic nature of trauma and the therapeutic potential of movement, rhythm, and sensory immersion (Csordas, 1994). This integrated framework provides a robust foundation for exploring rhythm- and nature-based interventions as holistic, trauma-responsive modalities that support both neurobiological regulation and emotional recovery.

### Search strategy

To identify relevant studies for the scoping review, a comprehensive search strategy was conducted, using the interdisciplinary Academic Search Complete, Ageline, Google Scholar, Medline, PsychInfo, and PubMed databases. The following search terms used to locate studies that focused on the combined effects of music and nature on mental health and wellbeing: “music” or “drum” or “drumming” or “taiko” AND “nature” or “garden” or “outdoors” or “horticulture” or “farm” AND “wellness” or “wellbeing” or “mental health.” These search words terms were also then repeated with “Global South” to prioritize research on the combined therapeutic use of music and nature in the Global South in particular, considering that the initial search results yielded results only from the Global North, however, this added no new search results. The search terms were used in combination using “AND” and “OR” to ensure a comprehensive search that helped identify studies that explored the intersection of music, nature, and mental health.

### Inclusion and exclusion criteria

To refine the search to ensure relevant literature was included, specific inclusion and exclusion criteria were applied. The inclusion criteria were designed to identify studies that focused on original peer-reviewed research with a direct connection to music, nature, as well as to wellbeing, and with this, mental and behavioral health and wellness-related outcomes. The inclusion criteria were that articles had to be (a) primary peer-reviewed research published in the English language (b) that involved both music and nature in the intervention, and (c) discussed how (if at all) wellbeing, and with this, mental health and/or behavioral health may have been impacted by the intervention(s) discussed in the reviewed studies.

The exclusion criteria were used to remove studies that did not align with the focus of this scoping review and/or studies that did not involve primary research. As such, excluded were (a) manuscripts that were not peer-reviewed, (b) studies that did not involve both nature-based and music-related therapeutic activities, (c) studies that did not involve primary research methods or analysis, and (d) studies that did not provide a clear discussion on the potential impacts on wellbeing, and with this, potential mental health or behavioral health outcomes, as a result of participating in nature-based and music related therapeutic activities.

### Case selection process

**FIGURE 1:**
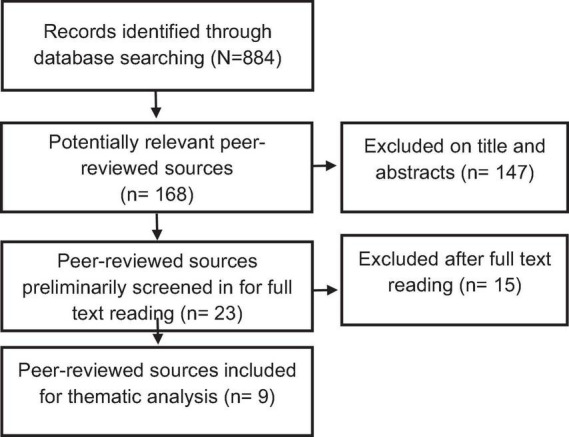
Scoping review results (modified from PRISMA and Hengelaar et al., 2018).

There were several stages during the study selection process to ensure only relevant studies were included in the final review. Initially, the search identified 884 potentially relevant cases. After filtering the potentially relevant cases for peer-reviewed manuscripts owing to our focus on primary peer-reviewed research, the number of potentially relevant cases was then reduced to 168 peer-reviewed articles. Upon reviewing the titles and abstracts of these manuscripts, 23 of these peer-reviewed articles met the inclusion criteria and were preliminarily screened in for full text reading. After fully reading these 23 potentially relevant articles, it was clear that 15 articles did not meet the search criteria, namely due to only including music or nature in the intervention without this being clear upon title and abstract review. Thus, 15 of these 23 articles were screened out, resulting in eight studies meeting the inclusion criteria upon full text review. A flowchart of this process is provided below in Figure 1.

### Critical appraisal

After the articles were fully read, the Caldwell et al. (2011) critical appraisal framework was applied to assess the quality of the reviewed studies. As such, each study was reviewed in-depth and compared, then rated from zero to two points with regard to trustworthiness and authenticity (Hand, 2020; Hand et al., 2022). The original version of the Caldwell et al. (2011) critical appraisal framework lists 18 questions regarding trustworthiness and authenticity, rated from zero to up to two points, for a total score of up to 36 points (Boyle, 2017; Hand, 2020). An abbreviated version was used to assess the quality of the reviewed research, as the first three questions were not useful for screening articles in for thematic analysis. Thus, each article could be scored up to 30 points in total using the same questions applied by Hand (2020).

Scores of 90% of the available 30 points or greater, beginning with 27 or more points indicated high quality research articles. Scores between 75% and 90%, or between 22.5 and 27 of the available 30 points, indicated that the articles were of moderate quality. Scores of less than 75%, or under 22.5 points, signified low quality, and it was determined that scores of under 60%, or less than 18 points would not be thematically analyzed, following the protocol described by Hand (2020); Hand et al. (2022).

### Thematic analysis

A thematic analysis was conducted by the first and third authors to offer an in-depth understanding of what is currently known about therapeutic interventions that include both nature-based and music-based components, based on primary published research that explored their effects on wellbeing, and thus, mental health and behavioral health outcomes. Following the steps for thematic analysis that have been described by Braun and Clarke (2006), the first author and third authors read and re-read the manuscripts as well as notes taken while reading the manuscripts to ensure familiarization with the data, then identified initial descriptive codes. Coding took place within an Excel sheet as well as within a Word document, to assist with organization. These codes were combined into categories, which were then reviewed, revised, and developed into distinct themes. The resulting themes were further refined and defined and are summarized in the following sections.

## Results

The final selection for the scoping review includes eight original research studies out of the 23 peer-reviewed articles that initially met the inclusion criteria which include a diverse range of approaches to exploring the intersection of music, nature, and their impacts on wellbeing, mental and behavioral health. In particular, four overarching themes were identified: (a) music- and nature-based activities yield benefits across various aspects of wellbeing, (b) multiple activities can be combined and adapted for diverse contexts and populations, (c) more research is needed on the combined therapeutic use of music and nature, and (d) choice and expression should be prioritized, which music and nature can facilitate. The critical appraisal results indicated that all of the reviewed articles were of moderate to high quality (with scores ranging from 22.75 to 28 as shown in Table 1), thus, all of the reviewed articles were thematically analyzed.

**TABLE 1:** Summary of key findings and recommendations among the reviewed articles.

No.	References	Method, *n*, and location	Key findings	Recommendations	Appraisal score
1	Adams and Beauchamp (2019)	Longitudinal qualitative cohort study in rural Wales over a 2 years period with six classes of children from primary schools (*N* = 187) ages 7–11 and their teachers. The students created musical ceremonies in groups within nature-based spaces. The interviews with the students and their teachers explored how creating music in nature affects spiritual, emotional, and education for youth.	The participants reported a heightened sense of reality and stronger connection to nature while making music while in nature. The intervention provided a heightened state of being or spiritual moments. Participants reported a sense of unity with people and the nature. This increased wellbeing during the music ceremonies, and long after.	Opportunities to create music in nature can offer a beneficial experience that does not typically occur in daily life. Future research should seek to explore how nature immersion and creating music can better promote childhood development and growth. Spiritual moments can be essential for a child’s development; making music in nature can offer this experience.	22.75
2	Arbuthnott and Sutter (2019)	Quantitative surveys in a two-part study using the 21-item Nature Relatedness Scale to measure connection to nature in Saskatchewan, Canada;15 high school students completed songwriting camps in nature and 23 completed classroom-only songwriting to examine whether songwriting in nature could increase connection with nature, mood, and/or creative reasoning. Study 2 involved two camps for 11 adults; results were compared with the adolescents and adults from a camp, to determine whether effects were linked with songwriting in nature.	Both students in the nature-based camps and in the classroom showed improved mood and connection with nature, and decreased negative emotions and stress. Compared with the control, all camp participants experienced greater connections with nature. The camps improved mood, creative reasoning, and nature connection for adults and adolescents, and elevating emotions. Songwriting improved creative reasoning, but it was unclear if the setting affected this.	Creating music in nature-based settings can offer a positive experience and greater connection with nature while experiencing music. Opportunities for both musical creative expression and nature immersion can enhance wellbeing. Nature-based education can improve connections with nature as well, while songwriting improves creative reasoning. The results suggest that creating music in nature-based settings can be adapted to fit into daily life and can also be used to address anxiety and improve mood.	27.5
3	Finnanger-Garshol et al. (2022)	Quantitative observational study in Norway with 88 adults in 17 adult day centers to compare standard vs. farm-based adult care services (FDCs) for people living with dementia. Mood was used to assess for emotional wellbeing. The Maastricht Electronic Daily Life Observation-tool was used to log aspects of daily life (mood, activity, physical effort, social interaction, engagement and location). Eleven kinds of activities were observed (sitting; eating/drinking, reading; quiz, music and spiritual; walking outdoors; exercise and dancing; self-care; social; domestic/cooking; farming; and unobservable/other activities).	While participants indicated a positive view of both farm-based and standard adult day programs, the FDCs were linked with higher rates of emotional wellbeing. Exercise, dance, quiz, music-based and spiritual activities were significantly linked with greater emotional wellbeing. These specific kinds of activities and social interaction were positively linked with emotional wellbeing regardless of facility type. Outdoor and farm-based activities were not linked with emotional wellbeing despite earlier research linking emotional wellbeing with horticultural-based activities.	Given that farm-based adult day programs are linked with higher rates of positive emotional wellbeing, standard adult day centers should incorporate aspects from farm-based settings to improve services. Providing opportunities for social interaction is especially important for emotional wellbeing. Future research is needed on best practices for fostering social interactions at adult day centers for people living with dementia. Music, quiz, and spiritual activities, exercise, and/or dancing could promote such social interactions, including in FDCs.	27
4	Kumpulainen et al. (2025)	Quantitative randomized acute cross-over study with 53 adults in Finland to compare the effects of listening to natural vs. urban soundscapes on psychophysiological wellbeing and relaxation for healthy adults, who listened to natural and urban soundscapes using headphones for 10 min each. Heart rate variability was the primary outcome. Heart and respiratory rates were also measured, and questionnaires were also completed, on wellbeing, creativity, and belonging.	Listening to the nature-based soundscape involved several benefits, including a lower heart and breathing rate, heightened positive emotions, feelings of belonging, less anxiety and depression, and increased comfort and enthusiasm, all which were more significant than the benefits of an urban-based soundscape. These effects indicate relaxation and recovery, which can be used to promote wellbeing.	The authors suggest that audio recordings can provide connections to nature for many people; the results are similar to studies involving full nature immersion. This intervention can be incorporated into future therapeutic practice (e.g., in mental health settings) owing to its accessibility and immediate benefits. Still, future research is needed with adults living with health conditions. And long-term effects should be studied.	27.25
5	Meyer et al. (2023)	Mixed methods study in Australia, to assess a 24 weeks program in a long-term care home among 33 veterans living with cognitive impairments or dementia who opted to exercise, engage with music therapy, combined reminiscence therapy, or sensory modulation, which included sensory garden walks. Questionnaires on cognitive impairment; depression; mobility; performance in daily activities; responsive behaviors; and quality of life were completed before and after the program, levels of engagement were assessed using field notes, and six interviews were conducted with staff and volunteers.	Allowing for choice in activities resulted in significant improvement across all measures regardless of the activity; physical and cognitive functional capacity, and depressive symptoms improved. The rate of falls substantially declined in the exercise group. Music therapy was supported by staff, family, and veterans. Activities continued beyond formal sessions; staff walked outdoors with clients while in dialogue, pausing to smell flowers, engaging in reminiscence, exercise, and sensory modulation.	The program and sensory modulation training enhanced staff confidence in modifying activities to meet unique needs. Staff and veterans enjoyed the music-based activities, and walked outdoors, engaged with nature and reminisced outside of the sessions, highlighting that management and staff can encourage engagement. Trauma-informed principles could inform future sensory modulation for older veterans. The improvements across measures were attributed to choice and expression, which should be prioritized in similar programs.	26.5
6	Pfeifer et al. (2019)	Quantitative experimental study in Germany with 84 university students. A Depth Relaxation Music Therapy (DRMT)/Hypnomusictherapy (HMT) session guided by a music therapist in a garden was compared with a seminar on silence (control group). The intervention and control were followed by a 6 min-and-30 s period of silence; relaxation, perception of time and self/space, and boredom were then assessed.	DRMT/HMT in a city garden followed by silence resulted in perceiving that more time had passed at a slower rate compared to the control. A slight correlation was found between relaxation and the perception of one’s body, indicating positive effects from meditation. The control was effective for relaxation, time perception, and presence; DRMT/HMT strengthened these experiences.	DRMT/HMT with silence in nature can be a useful low-cost technique to address these issues in a therapeutic manner. Future research should include the perception of time as a measure for future interventions, as an indicator of a meditative state which is linked with deep relaxation. Future studies should expand beyond university students, as this intervention can be adapted for a diverse range of populations.	27
7	Suko et al. (2025)	Quantitative crossover design with 69 participants between two universities in Finland and Japan to examine the restorative effects of natural sounds vs. music. Participants were split into two groups based on preferences for using music as mood regulation. The first group listened to natural sounds for 7 days (for 10 min per day), and their preferred music the second week. The second group did this in reverse order.	Participants who had not previously used music to regulate moods found both music and natural sounds restorative. Natural sounds were linked with consistent improvements in restorative experiences along with less stress. Participants who used music for mood regulation did not show a difference in the restorative experience. This group showed decreased stress when listening to natural sounds vs. music, with lower stress being slightly more significant when listening to natural sounds.	Both natural sounds and music can offer restorative effects and lower perceived stress. Yet, natural sounds are more effective. The authors suggest that utilizing natural sounds as a replacement for exposure to nature can be a cost-effective way to promote restoration and lower stress for those without access to nature. Natural sounds can enhance wellbeing in public spaces and may be incorporated into therapeutic activities. In everyday life, natural sounds may replace time in nature when time in nature is not possible. This can offer an escape from stress.	28
8	Witten et al. (2023)	Quantitative randomized control trials in England with 149 adults between 18 and 83 years of age with access to a computer, tablet or mobile device. Three different interventions were used in this study; this included seeing 25 nature related images, listening to 25 sounds that match the nature-based images, and combining both the sounds and nature-based images. The purpose of this study was to understand the potential effects on the participants’ mood.	For all three groups, decreased negative affect and depressive moods were found, along with an increased serenity affect. The results suggest that viewing nature-based images, listening to nature sounds, and combining both, were each able to increase feelings of serenity while decreasing negative affect and depressive moods. Still, participants with symptoms of depression and/or anxiety showed more significantly positive changes.	For individuals using mental health services, incorporating images and sounds (e.g., music) that resemble nature can improve mood. The authors suggest that a connection with nature can be obtained digitally with no physical presence in nature. This intervention can be useful for serving people living with depression and/or anxiety. Still, further research is needed on the combined use of nature-based images and music.	27.25

Of the eight articles screened in for review and thus, thematic analysis, the majority were quantitative in nature (Arbuthnott and Sutter, 2019; Pfeifer et al., 2019; Finnanger-Garshol et al., 2022; Witten et al., 2023; Kumpulainen et al., 2025; Suko et al., 2025). These quantitative studies mostly involved using questionnaires (Arbuthnott and Sutter, 2019; Witten et al., 2023; Suko et al., 2025) or observations to assess mood (Pfeifer et al., 2019). Still, other aspects of wellbeing were also assessed, such as pertaining to mental health, by evaluating stress levels (Arbuthnott and Sutter, 2019; Suko et al., 2025) as well as anxiety and depression (Witten et al., 2023; Kumpulainen et al., 2025), as well as relaxation (Kumpulainen et al., 2025; Pfeifer et al., 2019), and physiological responses, or specifically heart-rate variability (Kumpulainen et al., 2025). Further, one study involved observations at farm-based and traditional adult day programs (using the Maastricht Electronic Daily Life Observation-tool, MEDLO), to log aspects of daily life, such as mood, activity, physical effort, social interaction, engagement and location (Finnanger-Garshol et al., 2022).

In addition, one of the reviewed studies was qualitative, involving individual interviews to explore how creating music in nature affects spiritual, emotional, and education for youth (Adams and Beauchamp, 2019). Another study involved both quantitative and qualitative analyses, including both questionnaires to assess cognitive impairment, depression, performance in daily activities, mobility, responsive behaviors, and quality of life, as well as interviews with staff and volunteers (Meyer et al., 2023).

All of the reviewed studies were published research that was conducted outside of the United States (Adams and Beauchamp, 2019; Arbuthnott and Sutter, 2019; Pfeifer et al., 2019; Finnanger-Garshol et al., 2022; Meyer et al., 2023; Witten et al., 2023; Kumpulainen et al., 2025; Suko et al., 2025). Specifically, the studies took place in Australia (Meyer et al., 2023), Canada (Arbuthnott and Sutter, 2019), England (Witten et al., 2023), Finland (Kumpulainen et al., 2025; Suko et al., 2025), and Japan (Suko et al., 2025), Germany (Pfeifer et al., 2019), Norway (Finnanger-Garshol et al., 2022), and in Wales (Adams and Beauchamp, 2019).

A variety of strategies were used to incorporate music- and nature-based activities within the interventions of the reviewed studies. Most the studies focused on the use of music-based interventions in nature-based settings (Adams and Beauchamp, 2019; Arbuthnott and Sutter, 2019; Pfeifer et al., 2019; Finnanger-Garshol et al., 2022). This included a variety of activities, such as creating musical ceremonies in outdoor rural spaces (Adams and Beauchamp, 2019), song-writing camps in a national wildlife area (Arbuthnott and Sutter, 2019), incorporating music and singing at farm-based (versus standard) adult day programs (Finnanger-Garshol et al., 2022), and participating in a Depth Relaxation Music Therapy (DRMT)/Hypnomusictherapy (HMT) session guided by a music therapist within a city garden.

One of these studies, by Finnanger-Garshol et al. (2022) also described an intervention that involved the possibility of participants participating in both music-based and nature-based activities that involved engaging with nature. More specifically, in the farm-based and traditional adult day programs the researchers observed, participants were provided opportunities to care for plants, interact with animals, participate in farm-based activities, to walk outdoors, and sing, dance, or otherwise engage with music as well as other types of activities (e.g., reading, religious or spiritual activities, exercise, cooking, and other activities) (Finnanger-Garshol et al., 2022). Similarly, Meyer et al. (2023) emphasized the prioritization of participant choice in meaningful activities and described an intervention at a long-term care facility that allowed participants to opt to engage in music therapy and/or formal and informal sensory garden walks that may involve smelling flowers, among other activities (e.g., namely also offering the possibility of combined reminiscence therapy and sensory modulation) (Meyer et al., 2023).

Two additional studies involved listening to nature-based soundscapes (Kumpulainen et al., 2025; Suko et al., 2025) and in one study, listening to nature-based soundscapes was compared with listening to music (Suko et al., 2025). Witten et al. (2023) provided a comparison of viewing nature-based images, listening to nature sounds, and combining both nature-based images and the use of sound, guided by extant research on music therapy.

The populations of interest varied as well across the reviewed studies, suggesting multiple opportunities for the incorporation of music- and nature-based therapeutic strategies tailored to the needs of different target populations. For example, Adams and Beauchamp, 2019) conducted their research on developing musical ceremonies in nature-based environments with six classes of children from primary schools ages 7–11, Arbuthnott and Sutter (2019) conducted research on songwriting in nature-based (versus classroom) settings with both adolescents in high school as well as with adults. Two studies were conducted with university students (Pfeifer et al., 2019; Suko et al., 2025). One study was conducted with adults with access to a computer, tablet or mobile device owing to exposure to nature-based images and sounds being completely online (Witten et al., 2023), and another study included only healthy adults (Kumpulainen et al., 2025). In addition, two studies were conducted with older adults living with dementia (Finnanger-Garshol et al., 2022; Meyer et al., 2023) or other cognitive impairments (Meyer et al., 2023).

A summary of key findings from the methods used within these studies, their populations of focus, further details about each of these studies, and implications for the combined therapeutic use of music- and nature-based activities are provided in Table 1. This information will be discussed below as well, in the following sections, along with the overarching themes that were identified from the thematic analysis.

### Music- and nature-based activities yield benefits across various aspects of wellbeing

A key finding from this study was that the combination of music- and nature-based therapeutic activities resulted in manifold benefits that attend to various aspects of wellbeing (Adams and Beauchamp, 2019; Arbuthnott and Sutter, 2019; Pfeifer et al., 2019; Finnanger-Garshol et al., 2022; Meyer et al., 2023; Witten et al., 2023; Kumpulainen et al., 2025; Suko et al., 2025). In particular, the studies suggest that immersive experiences with music and nature can result in improved emotional wellbeing (Arbuthnott and Sutter, 2019; Finnanger-Garshol et al., 2022; Meyer et al., 2023; Kumpulainen et al., 2025) as well as enhanced psychological (Kumpulainen et al., 2025), physical (Meyer et al., 2023), cognitive (Arbuthnott and Sutter, 2019; Meyer et al., 2023; Kumpulainen et al., 2025), spiritual (Adams and Beauchamp, 2019), environmental (Adams and Beauchamp, 2019; Arbuthnott and Sutter, 2019), and social wellbeing (Adams and Beauchamp, 2019; Arbuthnott and Sutter, 2019; Finnanger-Garshol et al., 2022). Still, social benefits were only noted for studies that involved nature-based settings (Adams and Beauchamp, 2019; Arbuthnott and Sutter, 2019; Finnanger-Garshol et al., 2022).

As Adams and Beauchamp (2019) summarized, the combined therapeutic use of music and nature resulted in experiencing happiness, joy, connection with nature and with other participants, as well as spiritual moments, experienced for example, as “wonder, awe and a sense of inner calm or peace” (p. 10). Immersion in nature was found to offer a beneficial experience that does not typically occur in daily life which can result in deep and greater connections with nature (Adams and Beauchamp, 2019). Enhanced creative reasoning skills (Arbuthnott and Sutter, 2019) was also found to be a benefit of combining both music and nature to connectedness and wellbeing. Still, similar to being in a nature-based setting or engaging directly with nature, listening to nature-based soundscapes also resulted in an enhanced sense of creativity (Kumpulainen et al., 2025).

Such benefits across various aspects of wellbeing were often achieved by offering multi-sensory experiences (Adams and Beauchamp, 2019; Arbuthnott and Sutter, 2019; Finnanger-Garshol et al., 2022; Kumpulainen et al., 2025; Meyer et al., 2023; Pfeifer et al., 2019), such as through calming sights (Adams and Beauchamp, 2019; Arbuthnott and Sutter, 2019; Witten et al., 2023) (e.g., through birdwatching, Arbuthnott and Sutter, 2019), smells (e.g., of flowers, Meyer et al., 2023), and tactile experiences (e.g., feeling the air around them, Adams and Beauchamp, 2019, or with plants or animals, Finnanger-Garshol et al., 2022) in addition to appreciating the use of sound (Adams and Beauchamp, 2019; Arbuthnott and Sutter, 2019; Finnanger-Garshol et al., 2022; Kumpulainen et al., 2025; Meyer et al., 2023; Pfeifer et al., 2019; Suko et al., 2025; Witten et al., 2023). Still, in one article, it was suggested that nature-based soundscapes alone could be as beneficial to participants as multisensory experiences in nature-based settings (Kumpulainen et al., 2025).

Participants commonly described improvements in mood (Arbuthnott and Sutter, 2019; Finnanger-Garshol et al., 2022; Kumpulainen et al., 2025; Suko et al., 2025; Witten et al., 2023) as well as improved symptoms of anxiety (Arbuthnott and Sutter, 2019; Witten et al., 2023) or depression (Witten et al., 2023). Feelings of inner calm, peace or serenity (Adams and Beauchamp, 2019; Suko et al., 2025; Witten et al., 2023) and/or relaxation were common responses to the therapeutic use of music- and nature-based strategies (Adams and Beauchamp, 2019; Kumpulainen et al., 2025; Pfeifer et al., 2019; Suko et al., 2025; Witten et al., 2023). Improvements in ability to influence changes in behavior among people living with dementia were described by caregivers in response to training on sensory modulation as well as to programming which included both music- and nature-based activities (Meyer et al., 2023).

From a theoretical standpoint, these outcomes reflect key principles from Polyvagal Theory (Porges, 2011), suggesting that sensory engagement, particularly with safe and rhythmic environments (e.g., with nature-based spaces or stimuli) can activate the parasympathetic nervous system to support feelings of safety, connection with nature, and relaxation. For example, improved mood, lowered anxiety and depression, and experiences of calm or serenity (e.g., Adams and Beauchamp, 2019; Suko et al., 2025; Witten et al., 2023) can be influenced by auditory and multisensory stimuli such as making music, smelling the flowers, and/or appreciating the sway of the trees while playing music.

Simultaneously, these findings reinforce Embodiment Theory (Csordas, 1994), which emphasizes the centrality of the body in the therapeutic experience. Participants in the reviewed studies described sensory immersion (e.g., touching plants, feeling the air, hearing music) that engaged them physically and emotionally (Adams and Beauchamp, 2019; Arbuthnott and Sutter, 2019; Finnanger-Garshol et al., 2022; Meyer et al., 2023; Witten et al., 2023). This kind of bodily awareness and active engagement (e.g., in singing, drumming, walking, dancing, and/or gardening) may support trauma recovery as well by restoring a sense of agency, groundedness, and presence in one’s own body, which are key tents of embodied healing.

### Multiple activities can be combined and adapted for diverse contexts and populations

A diverse variety of mix-and-match music and nature-based activities were described across the reviewed studies. For example, this included opportunities for engagement with flowers (Meyer et al., 2023) and other plants as well as with farm-based activities, interactions with animals (Finnanger-Garshol et al., 2022), and/or time spent outdoors in nature (Adams and Beauchamp, 2019; Arbuthnott and Sutter, 2019; Finnanger-Garshol et al., 2022; Meyer et al., 2023; Pfeifer et al., 2019). In one study, participants could also view nature-based images, which along with exposure to nature-based sounds, could be especially useful for people with limited mobility, green space access, and/or health issues that may prevent them from directly engaging nature (Witten et al., 2023). Participants were also offered opportunities to dance (Finnanger-Garshol et al., 2022), to listen to music (Finnanger-Garshol et al., 2022; Suko et al., 2025) or nature-based sounds or soundscapes (Kumpulainen et al., 2025; Suko et al., 2025; Witten et al., 2023) or to create music themselves using instruments (Adams and Beauchamp, 2019) or by singing (Arbuthnott and Sutter, 2019; Finnanger-Garshol et al., 2022). Participants could experience a Depth Relaxation Music Therapy (DRMT)/Hypnomusictherapy (HMT) session in a nature-based setting (Pfeifer et al., 2019) or engage in music therapy (Meyer et al., 2023). Moreover, it is noteworthy that activities could take place in formal therapeutic sessions or informally with staff (Meyer et al., 2023).

The music- and nature-based strategies discussed above and their combined use were effective at improving emotional wellbeing and relaxation as well as multiple other benefits across several dimensions of wellbeing as discussed above, with a variety of populations, which is promising. The populations of focus for the reviewed studies ranged from children from 7 to 10 years (Adams and Beauchamp, 2019) to adolescents who were still in high school to adults (Arbuthnott and Sutter, 2019; Witten et al., 2023), such as young adults in universities (Pfeifer et al., 2019; Suko et al., 2025), as well as older adults living with dementia (Finnanger-Garshol et al., 2022; Meyer et al., 2023). The combined use of music and nature-based interventions were also found to be effective among veterans (Meyer et al., 2023) and civilians (Arbuthnott and Sutter, 2019; Finnanger-Garshol et al., 2022; Kumpulainen et al., 2025; Pfeifer et al., 2019; Suko et al., 2025; Witten et al., 2023) alike, as well as with adults living with cognitive impairments (Meyer et al., 2023).

A diverse variety of nature-based settings was also described within the reviewed studies for consideration in future music- and nature-based therapeutic programming. This involved of music-based interventions like music making (Adams and Beauchamp, 2019; Arbuthnott and Sutter, 2019), singing and engaging with music (Finnanger-Garshol et al., 2022), and a Depth Relaxation Music Therapy (DRMT)/Hypnomusictherapy (HMT) session (Pfeifer et al., 2019) being implemented in nature-based settings ((Adams and Beauchamp, 2019; Arbuthnott and Sutter, 2019; Finnanger-Garshol et al., 2022; Pfeifer et al., 2019). The nature-based spaces themselves also varied, to include outdoor rural (Adams and Beauchamp, 2019) or wildlife areas (Arbuthnott and Sutter, 2019), farm-based settings (Finnanger-Garshol et al., 2022), and one study took place in a garden (Pfeifer et al., 2019). Walking in gardens was also described as a potential activity that participants could select in one study (Meyer et al., 2023).

The combination of music and nature can be adapted to fit into daily life and into multiple different kinds of interventions (Arbuthnott and Sutter, 2019). For example, it was noted that nature-based music can be incorporated into future mental health practice as an accessible intervention with immediate benefits (Kumpulainen et al., 2025). Similarly, it was suggested that both nature-based images and soothing sounds should be incorporated into interventions that influence behavior, with potential to improve mental health and wellbeing (Witten et al., 2023). Further, it was recommended that standard adult day programs should incorporate aspects of farm-based programs (e.g., horticulture-based activities, such as the ability to care for plants and ability to walk outdoors) while also offering opportunities to engage with music to improve emotional wellbeing among older adults living with dementia (Finnanger-Garshol et al., 2022). Music and nature-based activities are cost effective as well (Meyer et al., 2023; Suko et al., 2025; Witten et al., 2023), which can further facilitate the application of these music- and nature-based activities in a variety of contexts and interventions.

### Choice and expression should be prioritized, which music and nature can facilitate

The ability of music- and nature-based interventions to offer opportunities for choice and the role of choice in the success of such programs were highlighted as well (Adams and Beauchamp, 2019; Arbuthnott and Sutter, 2019; Meyer et al., 2023). For example, participants could choose to work in groups or not when creating music (Arbuthnott and Sutter, 2019). Participants could also choose their musical instruments and could create a musical ceremony of their choice (Adams and Beauchamp, 2019). Further, participants could choose to participate in one or more activities, some of which were music- or nature-based, being mindful of differing abilities and preferences (Meyer et al., 2023).

This emphasis, on choice and adaptability, also aligns with principles of person-centered practice (Meyer et al., 2023). As Meyer et al. (2023) shared, offering participants choice over how they would express themselves, such as in music therapy and/or during nature walks and in during other sensory activities allowed staff to get to know participants better. As such, staff derived more meaning from their work with older veterans living with dementia or cognitive impairments owing to the ability to form stronger connections with participants due to multiple opportunities for participant choice and self-expression (Meyer et al., 2023).

Moreover, Meyer et al. (2023) reported that in their study, improvements were observed across all outcomes (with regard to cognitive impairment, depressive symptoms, performance in daily activities, mobility, responsive behaviors, and quality of life). This success was specifically attributed to the opportunity for choice as well as to opportunities for self-expression (Meyer et al., 2023). Meyer et al. (2023) highlighted that “older people wish to feel recognized and valued, and have opportunities to maintain and form reciprocal relationships,” noting that while there may be challenges involved with allowing for choice in research, this flexibility helped staff to ensure more tailored and focused interactions with participants (p. 912).

Thus, while only three of the eight reviewed studies emphasized the importance of choice when implementing music- and nature-based interventions and the ability of music- and nature-based interventions to facilitate further choices for self-expression (Adams and Beauchamp, 2019; Arbuthnott and Sutter, 2019; Meyer et al., 2023), this finding was especially noteworthy. This presents potentially far-reaching person-centered and trauma-informed implications, as providing safety and choice while elevating unique strengths and abilities (also described as empowerment) are critical components of trauma-informed care (Meyer et al., 2023). Accordingly, allowing for choice and flexibility in music- and nature-based programming and research could help enhance work multiple populations who have experienced trauma, ranging from childhood (Adams and Beauchamp, 2019) into adulthood (Arbuthnott and Sutter, 2019) and well into later life (Meyer et al., 2023).

### More research is needed on the combined therapeutic use of music and nature

Further research is needed to explore how the use of both music and nature can be combined in therapeutic interventions (Adams and Beauchamp, 2019; Arbuthnott and Sutter, 2019; Pfeifer et al., 2019) for a variety of populations in multiple settings and contexts (Pfeifer et al., 2019). In particular, Adams and Beauchamp (2019) highlighted that research is needed on how music and nature can be used to promote childhood development and growth. Arbuthnott and Sutter (2019) also suggested that future research could benefit from an exploration of the vulnerability involved in performing original songs in a nature-based environment for peers.

Research is needed on best practices for promoting social interactions in adult day programs for people living with dementia, to include farm-based adult day programs as well (Finnanger-Garshol et al., 2022). Music-based activities can be used to facilitate such social interactions (Finnanger-Garshol et al., 2022). Finnanger-Garshol et al. (2022) also recommended that aspects of farm-based day settings should be studied to learn more about how they are linked with emotional wellbeing as well as how aspects of farm-based adult day activities and settings could be applied in other care contexts.

Additionally, it was noted that research on the use of nature-based music or soundscapes is needed with adults living with health conditions, and the long-term effects of nature-based music or soundscapes should be studied (Kumpulainen et al., 2025). More research is needed on DRMT/HMT with silence incorporated as well, in nature-based versus clinical environments (Pfeifer et al., 2019). It was also recommended that the potential benefits of blue spaces (e.g., marked by large bodies of water such as lakes and/or fountains) should be explored given that the restorative benefits of green spaces (marked by areas mostly covered in plants, grass, trees, etc., such as parks, farms, and gardens) have been established as appropriate settings for music-based interventions (Suko et al., 2025).

## Discussion

The purpose of this scoping review was to explore what is known according to current peer-reviewed primary research about the combined therapeutic use of music and nature as well as the potential effects of such interventions on overall wellbeing, particularly on mental and behavioral health outcomes. Four themes were identified across the eight articles that were reviewed. These included: (a) music- and nature-based activities yield benefits across various aspects of wellbeing, (b) multiple activities can be combined and adapted for diverse contexts and populations, (c) more research is needed on the combined therapeutic use of music and nature, and (d) choice and expression should be prioritized, which music and nature can facilitate. The reviewed studies demonstrated benefits for a diverse variety of populations, including children (Adams and Beauchamp, 2019) and adults (Arbuthnott and Sutter, 2019), including adults living with dementia (Finnanger-Garshol et al., 2022; Meyer et al., 2023) and cognitive impairment (Meyer et al., 2023).

Numerous benefits were found as a result of engagement with both music and nature across multiple dimensions of wellbeing. Synergistically summarizing these benefits not only offers an overview of the current available primary research on the combined therapeutic use of music and nature for improving wellbeing, but the results of this scoping review also move beyond the initial reviewed study findings, such as by underscoring the diverse aspects of wellbeing that were enhanced. As discussed in the results section, this included enhanced emotional and psychological wellbeing, physical wellbeing, as well as greater spiritual, environmental, and spiritual wellbeing following participation in both therapeutic music- and nature-based interventions.

While most of the reviewed studies resulted in improved mood and overall wellbeing, particularly emotional wellbeing after engagement in music- and nature-based therapeutic activities, there were variations in the foci and approaches used within the reviewed studies as well as variations in the specific activities that were described. For example, some of the reviewed studies focused on outdoor education and musical creativity, while others explored the mood effects of nature-related stimuli. Along with the authors’ recommendations and discussion of potential implications based on their research findings, this variation in context and activities suggests possibilities for integrating music in nature in daily life as well as within interventions to improve wellbeing, including with regards to mental and behavioral health.

In line with this, Polyvagal Theory and Embodiment Theory offer compelling frameworks to better understand how these multisensory, choice-based, and rhythmically engaging activities may promote neurophysiological regulation and embodied healing. Such activities can activate the parasympathetic nervous system and support trauma recovery through embodied engagement and co-regulation (Csordas, 1994; Porges, 2011). Polyvagal Theory and Embodiment Theory can not only help explain the mechanisms behind observed outcomes but also emphasize the accessibility and adaptability of these interventions for diverse populations.

As discussed, in alignment with the Biophillia Hypothesis, the results suggest that music-making in natural settings can foster deep connections with nature, evoke biophilic responses, and lead to transformative spiritual experiences. Engaging in music-based activities in outdoor environments also seems to boost mood, creativity, and improve overall wellbeing. These findings align with existing literature regarding the effects of using music and nature independently. For example, music has been shown to increase emotional responses, reduce stress, and improve mood (Rebecchini, 2021), while the therapeutic use of nature has also been shown to decrease anxiety, reduce depressive symptoms, and increase feelings of relaxation and calmness (Jimenez et al., 2021). In the reviewed studies, the combination of these elements offers a synergistic multi-sensory approach that allows for further prioritization of choice and adaptability, which can be tailored to a variety of populations, including for people living with disabilities (Meyer et al., 2023) and/or with dementia (Finnanger-Garshol et al., 2022; Meyer et al., 2023) and adults (Arbuthnott and Sutter, 2019; Finnanger-Garshol et al., 2022), including adults living with dementia (Finnanger-Garshol et al., 2022; Meyer et al., 2023) and cognitive impairment (Meyer et al., 2023).

Numerous benefits were found as a result of engagement with both music and nature across multiple dimensions of wellbeing. Synergistically summarizing these benefits not only offers an overview of the current available primary research on the combined therapeutic use of music and nature for improving wellbeing and with this, mental and behavioral health, but the results of this scoping review also move beyond the initial research findings (Butler et al., 2016). For example, the results of this scoping review underscore the multiple aspects of wellbeing that were enhanced following participation in both therapeutic music- and nature-based activities as reported by the reviewed manuscripts. As discussed above, this included enhanced emotional and psychological wellbeing, physical wellbeing, as well as with regards to spiritual, environmental, and spiritual wellbeing.

While findings are somewhat similar across the reviewed studies, which largely reported improved mood and overall wellbeing, particularly emotional wellbeing following participation in music- and nature-based therapeutic activities, there were variations in focus and approach between the studies and along with this, variations in the specific activities that were described. For example, some of the reviewed studies focused on outdoor education and musical creativity, while others explored the mood effects of nature-related stimuli. Along with the authors’ recommendations and discussion of potential implications, this variation suggests possibilities for integrating music in nature in daily life as well as within interventions to improve wellbeing, and with this, mental and behavioral health, as discussed in the results section as well as below.

### Implications for future practice and programming

The positive outcomes observed in the reviewed studies suggest several practical applications for integrating music and nature which could offer both children and adults the chance to connect with nature and socially, with one another. Along with listening to and/or making music, exposure to nature-related stimuli such as being outside in green spaces or interacting with plants could also be effectively reduce negative affect and depressive symptoms.

This presents an opportunity for mental health clinicians to explore visual- and auditory-based strategies to promote greater connection with nature and improved emotional wellbeing as part of future therapeutic interventions and programming. Thus, overall, the results highlight the potential for integrating both music- and nature-based interventions into wellness programs and mental health programming. While music- and nature-based activities can be used to offer more opportunities for choice and flexibility owing to the nature of these approaches, it is also recommended that future programming should prioritize choice in the kinds of music- and/or nature-based activities clients engage in, to reflect a person-centered and trauma-informed approach (Meyer et al., 2023).

### Implications for future research

Several opportunities and recommendations for future research were identified by the authors of the reviewed studies. Most prominent was the need for more research on the combined therapeutic use of music and nature to promote greater wellbeing and with this, enhanced mental and behavioral health. This pressing need for research was evidenced both in the thematic analysis and by the limited available research on the combined therapeutic use of music and nature. Further comprehensive studies are especially needed to explore the combined effects of music and nature-based interventions that explore the underlying mechanisms of these outcomes. For example, further research should explore how music and nature each uniquely contribute to emotional wellbeing (e.g., mood) in comparison to how the combined use of these strategies can enhance wellbeing. Future research on music- and nature-based interventions should include larger sample sizes and longer longitudinal studies to determine the long-term effects of these interventions.

Further research is also needed to investigate the potential impact of different styles of music and different types of nature-based settings on mental health and wellbeing (e.g., actively developing original music with instruments versus singing, or passively listening to music, Arbuthnott and Sutter, 2019 in green spaces versus blue spaces, Suko et al., 2025). Participating in music-based interventions while in natural spaces versus in addition to actively engaging with nature, such as with plants, through gardening and/or other nature-based therapeutic practices (e.g., therapeutic terrarium making) also merits further research. Exploring the cultural and social aspects of music- and nature-based interventions could offer a more nuanced understanding of their potential role in mental health as well. For example, As Adams and Beauchamp (2019) have explained, several indigenous cultures ascribe to a biophilic perspective, maintaining that people are part of the natural environment around them, just as plants and animals are, which may explain the deep connections participants described feeling with nature after making music in natural spaces. Thus, further investigation into biophilic responses to nature among different cultures and exploring which kinds of music and nature-based strategies may be most culturally appropriate for different populations is warranted. Meanwhile, this scoping review highlights the benefits of combining nature and music while shedding light on the need for further research to better understand their unique and combined effects as well as to identify new opportunities for practical music- and nature-based applications in both clinical and natural settings.

## Limitations

In this scoping review, peer-reviewed research was prioritized, to offer an overview of what is currently known about the combined use of music and nature according to high-quality primary research. Still, future research that includes such gray sources in efforts to find additional studies may be warranted, such as research, theses and dissertations in addition to peer-reviewed research. Further, the studies that were reviewed were all published in English, as this is the only language the authors can read fluently and thus, assess. However, this may have limited the results of the scoping review. While as discussed in the results section, the refined search strategy reflected attempts to prioritize research from underrepresented perspectives from the Global South in particular, no research on the combined therapeutic use of music and nature was found in the Global South, and no research on the combined therapeutic use of music and nature had been found in the US either. All of the screened in studies were published in Australia, Canada, in Western Europe, and in Japan. Thus, further efforts are needed to conduct and synthesize research on the combined therapeutic use of music and nature for mental health and wellbeing in the Global South in particular as well as in the United States, prioritizing cultural practices and perspectives.

## Conclusion

This scoping review investigated what is known about the combined therapeutic use of music and nature based on primary peer-reviewed research, as well as how interventions incorporating both music and nature may be used to potentially improve wellbeing and with this, mental and behavioral health. Eight original research articles were identified after a comprehensive search process that focused on the combined effects of music and nature and their impact on wellbeing, as well as on mental health and behavioral health outcomes. The findings from the reviewed studies consistently revealed positive results, suggesting that integrating music and nature can be used to improve mood and emotional wellbeing as well as to potentially decrease anxiety, stress, and depressive symptoms.

The results underscore the need for further research to explore the potential impact of the combined therapeutic use of music and nature and how integrating these strategies may be used to promote wellbeing and social interaction in particular while prioritizing choice and adaptability to meet unique needs. Future research could contribute to the development of accessible, adaptable, and innovative strategies that recognize the potential of both music and nature to be used to improve emotional wellbeing as well as mental and behavioral health outcomes, such as by offering further opportunities for choice and self-expression. One size does not fit all—there are clients who may prefer time interacting with nature and/or time in nature-based settings (e.g., parks or gardens) over creating or listening to music, and similarly, there are clients who may be more interested in music than nature. Integrating these low-cost and restorative kinds of interventions together and allowing for choice (e.g., of music versus nature-based activities, or both) can be used to better align with person-centered, accessible and trauma-informed practice.
