# Correction: Integrating music and nature: a scoping review of research on interventions involving both music- and nature-based strategies for mental health and wellbeing

**DOI:** 10.3389/fnhum.2026.1933990

**Published:** 2026-07-21

**Authors:** Frontiers Production Office

**Affiliations:** Frontiers Media SA, Lausanne, Switzerland

**Keywords:** music, nature, scoping review, qualitative, thematic analysis

In the section, **Results**, subheading *More research is needed on the combined therapeutic use of music and nature*, paragraph 4 is a verbatim duplication of paragraph 2. Paragraph 4 has therefore been deleted and the section now reads:

*More research is needed on the combined therapeutic use of music and nature*

Further research is needed to explore how the use of both music and nature can be combined in therapeutic interventions (Adams and Beauchamp, 2019; Arbuthnott and Sutter, 2019; Pfeifer et al., 2019) for a variety of populations in multiple settings and contexts (Pfeifer et al., 2019). In particular, Adams and Beauchamp (2019) highlighted that research is needed on how music and nature can be used to promote childhood development and growth. Arbuthnott and Sutter (2019) also suggested that future research could benefit from an exploration of the vulnerability involved in performing original songs in a nature-based environment for peers.

Research is needed on best practices for promoting social interactions in adult day programs for people living with dementia, to include farm-based adult day programs as well (Finnanger-Garshol et al., 2022). Music-based activities can be used to facilitate such social interactions (Finnanger-Garshol et al., 2022). Finnanger-Garshol et al. (2022) also recommended that aspects of farm-based day settings should be studied to learn more about how they are linked with emotional wellbeing as well as how aspects of farm-based adult day activities and settings could be applied in other care contexts.

Additionally, it was noted that research on the use of nature-based music or soundscapes is needed with adults living with health conditions, and the long-term effects of nature-based music or soundscapes should be studied (Kumpulainen et al., 2025). More research is needed on DRMT/HMT with silence incorporated as well, in nature-based versus clinical environments (Pfeifer et al., 2019). It was also recommended that the potential benefits of blue spaces (e.g., marked by large bodies of water such as lakes and/or fountains) should be explored given that the restorative benefits of green spaces (marked by areas mostly covered in plants, grass, trees, etc., such as parks, farms, and gardens) have been established as appropriate settings for music-based interventions (Suko et al., 2025).

The original version of this article has been updated.

